# Caffeine for apnea and prevention of neurodevelopmental impairment in preterm infants: systematic review and meta-analysis

**DOI:** 10.1038/s41372-024-01939-x

**Published:** 2024-03-29

**Authors:** Elizabeth A. Oliphant, Sara M. Hanning, Christopher J. D. McKinlay, Jane M. Alsweiler

**Affiliations:** 1 Department of Paediatrics: Child and Youth Health, University of Auckland, Auckland, New Zealand; 2 School of Pharmacy, University of Auckland, Auckland, New Zealand; 3 Kidz First Neonatal Care, Te Whatu Ora Counties Manukau, Auckland, New Zealand; 4 Starship Child Health, Te Whatu Ora Te Toka Tuamai, Auckland, New Zealand

**Keywords:** Outcomes research, Paediatrics, Translational research, Respiratory tract diseases, Neurodevelopmental disorders

## Abstract

This systematic review and meta-analysis evaluated the evidence for dose and effectiveness of caffeine in preterm infants. MEDLINE, EMBASE, CINHAL Plus, CENTRAL, and trial databases were searched to July 2022 for trials randomizing preterm infants to caffeine vs. placebo/no treatment, or low (≤10 mg·kg^−1^) vs. high dose (>10 mg·kg^−1^ caffeine citrate equivalent). Two researchers extracted data and assessed risk of bias using RoB; GRADE evaluation was completed by all authors. Meta-analysis of 15 studies (3530 infants) was performed in REVMAN across four epochs: neonatal/infant (birth-1 year), early childhood (1–5 years), middle childhood (6–11 years) and adolescence (12–19 years). Caffeine reduced apnea (RR 0.59; 95%CI 0.46,0.75; very low certainty) and bronchopulmonary dysplasia (0.77; 0.69,0.86; moderate certainty), with higher doses more effective. Caffeine had no effect on neurocognitive impairment in early childhood but possible benefit on motor function in middle childhood (0.72; 0.57,0.91; moderate certainty). The optimal dose remains unknown; further long-term studies, are needed.

## Introduction

Infants born preterm are physiologically and metabolically immature and have higher rates of morbidity and mortality, and poorer long-term neurodevelopmental outcomes than those born at term [1]. Amongst other issues, they are at risk of apnea of prematurity [2] and intermittent hypoxemia [3], which result in a decrease in oxygen saturation and bradycardia and have been associated with increased risk of neurodevelopmental impairment [4, 5]. Rates of apnea are correlated with the degree of prematurity, occurring most frequently in extremely preterm infants, though late preterm infants are also affected [2]. Late preterm infants also experience frequent episodes of intermittent hypoxemia [3] and poorer neurodevelopmental outcomes than term-born infants [6].

Methylxanthines are respiratory stimulants that have been used in preterm neonates for decades to both prevent and treat apnea of prematurity and to facilitate extubation [7]. Caffeine is a naturally occurring methylxanthine used extensively worldwide for hundreds of years for its central nervous system stimulant properties [7]. Caffeine and other methylxanthines, such as theophylline, have been used in the treatment of apnea in newborn infants since the 1970s [8]. The precise mechanism by which methylxanthines improve respiratory function continues to be debated, but caffeine is known to stimulate the respiratory center in the medulla by antagonizing adenosine A1 and A2A receptors, increasing sensitivity and response to carbon dioxide and PO_2_ and enhancing diaphragmatic function [9]. Caffeine is now used in preference to other methylxanthines due to its wider therapeutic window and longer duration of action in neonates, which allow for daily dosing and remove the need for therapeutic drug monitoring [10, 11].

Despite this longstanding clinical use there remain several evidence gaps, including indications for treatment, dosing regimen, the most appropriate patient population, and the short- and long-term effects of caffeine therapy [12]. The aim of this systematic review was to assess the effectiveness of caffeine in reducing the rate or occurrence of apnea and reducing long‐term neurodevelopmental impairment in preterm infants (<37 weeks’ post-menstrual age [PMA]). A secondary aim was to assess if there is any difference in these outcomes between caffeine given at standard doses (≤10 mg·kg^−1^ caffeine citrate equivalent) and high doses (>10 mg·kg^−1^ caffeine citrate equivalent).

## Methods

This systematic review was guided by the Cochrane Handbook for Systematic Reviews of Interventions [13] and is reported according to the Preferred Reporting Items for Systematic Reviews and Meta-Analyses (PRISMA) statement [14]. Prior to the literature search being conducted, the protocol was registered with the Prospective Register of Systematic Reviews (PROSPERO, CRD42020154678).

We included all randomized controlled trials (RCTs) in preterm infants (<37 weeks’ PMA) of caffeine (at any dose and for any reason) vs. placebo or no treatment (comparison one), or high-dose caffeine (>10 mg·kg^−1^ citrate equivalent) vs. low-dose caffeine (≤10 mg·kg^−1^ caffeine citrate equivalent) (comparison two), which reported one or more prespecified outcomes. We included published studies and those published in abstract if they included sufficient information to confirm eligibility and allow Grading of Recommendations Assessment, Development and Evaluation (GRADE) [15]. We did not include observational or non-randomized studies. No limit was placed on year of publication, and studies in any language were included and translated if an English abstract was available for the initial screening stage.

We reported outcomes across four developmental epochs: neonatal/infancy (<1 year of age), early childhood (ages 1–5 years), middle childhood (ages 6–11 years) and adolescence (ages 12–19 years). If longitudinal studies reported multiple assessments of an outcome within the epoch, the last reported assessment in each epoch was included in the analysis.

The primary outcome for the neonatal/infant epoch was apnea, defined as a pause in breathing of ≥20 s, or <20 s with bradycardia (heart rate <100 beats per minute [bpm]), cyanosis or pallor [16], or as per author definitions. For all other epochs, the primary outcome was neurocognitive impairment, defined by authors, using standardized tests appropriate for age.

Secondary outcomes for the neonatal/infant epoch included bronchopulmonary dysplasia (BPD), defined as ongoing requirement for oxygen or respiratory support at 36 weeks’ PMA; intermittent hypoxemia, expressed as events per hour and defined as a fall in oxygen saturation (SpO_2_) of 10% or more from baseline, or as defined by authors; retinopathy of prematurity (ROP) Stage III or worse [17]; intraventricular hemorrhage (IVH) grade III or IV [18]; patent ductus arteriosus (PDA), defined as use of medical or surgical treatment for ductal closure; tachycardia, defined as mean heart rate ≥160 bpm or as per authors; duration of mechanical ventilation; duration of positive pressure support; growth velocity, including weight gain (g.kg^−1^.day^−1^), linear growth (cm.week^−1^) and head growth (cm.week^−1^) to 36 weeks’ PMA (or as defined by authors); death; survival without neurosensory impairment (including, but not limited to deafness, blindness and cerebral palsy); and time to establish full enteral feeds (as defined by authors).

For all other epochs, secondary outcomes included: motor impairment, defined by authors using standardized tests appropriate for age; hearing impairment, defined as requiring one or more hearing aids or worse, or as per authors; visual acuity less than 1 LogMAR, or as per authors; death; survival without neurosensory impairment, including, but not limited to, deafness, blindness, death and cerebral palsy; emotional-behavioral difficulties, as defined by authors; cerebral palsy; chronic lung disease, defined as physician-diagnosed asthma or ≥2 episodes of parent-reported wheeze, or as per authors; and height and weight expressed as Z-scores.

### Search strategy

We searched Pubmed, Medline, Embase, the Cumulative Index to Nursing and Allied Health Literature (CINHAL Plus) and the Cochrane Central Register of Controlled Trials (CENTRAL) databases from inception to 11 July 2022 using relevant MeSH terms and keywords (caffeine and premature/ prematurity/ preterm/ low birthweight and variations). The search was limited to studies involving humans, with no limit on year of publication or language. No limits on study type were applied at the initial search stage. We also searched The World Health Organization International Clinical Trials Registry Platform (ICTRP) (who.int/ictrp/search/en/), the US National Library of Medicine Clinical Trials Registry (clinicaltrials.gov), and Australia and New Zealand Clinical Trials Registry (ANZCTR) (anzctr.org.au), for any additional trials meeting the inclusion criteria not located through the above searches. Where results of trials were not available in the public domain, we contacted the authors listed in the trial registration to confirm the status of the trial, and whether any results were available for inclusion. We hand-searched bibliographies of included studies, review papers and conference abstracts to identify any additional studies. Covidence (Covidence Systematic Review Software, Veritas Health Innovation, 2020) was used to manage search results and screen studies for inclusion.

### Study selection

Two review authors independently screened all retrieved titles and abstracts to assess eligibility for inclusion. The full text of all potentially relevant studies was retrieved and assessed independently by two authors to determine eligibility. Any disagreements were resolved by mutual discussion and consultation with a third author if required. Summary characteristics of each study were extracted and tabulated.

### Data extraction, bias, and quality assessment

Two authors independently extracted data from all included studies using a prespecified data form. Any discrepancies were resolved by mutual discussion and consulting a third author if required. Additional information was sought from study corresponding authors if information was unclear or not published.

Two review authors independently assessed the risk of bias (RoB) of all included trials using the Cochrane RoB tool [19] for the following domains: sequence generation (selection bias); allocation concealment (selection bias); blinding of participants and personnel (performance bias); blinding of outcome assessment (detection bias); incomplete outcome data (attrition bias); selective reporting (reporting bias); any other bias. Any disagreements were resolved by mutual discussion or consulting a third author if necessary. For one study, where EO, JA, and CM were investigators, an alternative independent colleague (AW) with no association to the study conducted the data extraction and RoB assessment in conjunction with SH.

Review Manager (RevMan version 5.4.1. The Cochrane Collaboration, 2020) was used to summarize and analyze the data. Meta-analysis using fixed effects was performed if data from >2 RCTs were available. Apnea was reported using different measures that precluded a single meta-analysis; therefore, apnea was analyzed both as a dichotomous and continuous variable. We calculated the risk ratio (RR) for dichotomous outcomes and mean difference (MD) for continuous outcomes, with confidence intervals (CI) of 95%. If data were reported as median and interquartile range, means and standard deviations were estimated [20]. Planned secondary analyses included subgroup analysis by indication for caffeine and gestation length. Statistical heterogeneity was defined as an *I*^2^ > 50% and low *p*-value for the Chi-Square test, and categorized according to GRADE guidelines [15]. Methodological causes of heterogeneity were explored via subgroup analysis and sensitivity analysis, excluding studies at high risk of bias.

Outcomes were classified by all authors according to their importance for decision-making using GRADE classifications (7–9 critical, 4–6 important but not critical, 1–3 less important) [15]. Certainty of the evidence was assessed using the GRADE framework [15] and agreed by all authors. Imprecision was assessed using optimal information size (OIS) assuming alpha 0.05 and beta 0.2 [21] and considered serious if the total number of participants was less than the OIS for the outcome, or very serious if total participants numbered less than half the OIS. For continuous outcomes we assumed alpha 0.05 and beta 0.2, and delta 0.33.

Study characteristics and results were tabulated, and forest plots generated for all comparisons where data was available.

## Results

### Literature search and study selection

**Fig. 1:**
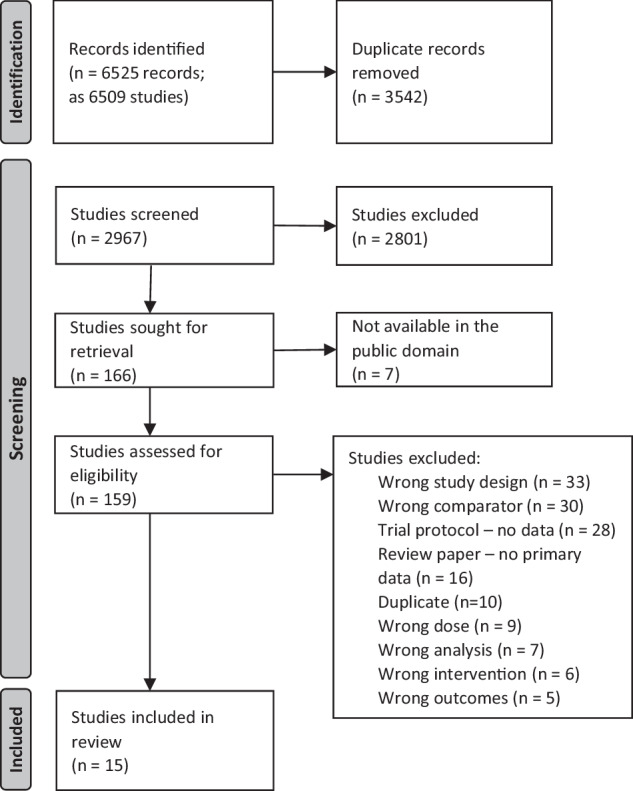
Preferred Reporting Items for Systematic Reviews and Meta-Analyses flow diagram of study selection.

Our search identified 6509 studies (Fig. 1). Following the removal of 3542 duplicates, 2968 studies were screened and 2801 excluded. The full text of 159 papers were reviewed, resulting in the inclusion of 15 studies in the final review.

### Study characteristics

We identified 15 eligible RCTs enrolling a total of 3530 premature infants. Most trials enrolled infants born at <32 weeks’ PMA [22–30], although some included infants up to 35 [31] or 36 [32] weeks, or defined eligibility based on birthweight [24, 33–35] or clinical decision to treat with caffeine [36]. Eight trials compared caffeine to placebo or no treatment [22–25, 31, 33–35]. Seven trials compared different doses of caffeine [26–30, 32, 36], including one [32] with four different dosing arms and a placebo arm, which contributed to both comparisons. Trials were widely geographically located and all except one [32] enrolled only infants in neonatal units. Most trials were small, with only one enrolling more than 300 infants [33] (Table 1). Eight of the included trials had high RoB in one or more domains [24, 25, 28, 30–32, 34, 35], especially for ‘incomplete outcome data’ (Table 2). All included studies reported at least one outcome for the neonatal/infant epoch. Two studies [33, 36] reported outcomes in early childhood, and only one study [33] reported outcomes in middle childhood. No studies reported results in adolescence.

**Table 1:** Study characteristics.

Study	Country	Eligibility	Enrolled (*n*)	Intervention	Comparator^a^	Primary Outcome(s)^b^	Notes
Comparison 1: caffeine vs placebo / no treatment							
Armanian [35]	Iran	Premature infants BW ≤ 1200 g Spontaneous breathing at 24 h of life.	52	*Loading:* IV “caffeine” (salt not specified) 20 mg/kg loading dose on first day of life	*Loading & Maintenance:* Equivolume doses of IV saline 0.9%	Apnea Bradycardia Cyanosis	If infants in the control group demonstrated apnea, caffeine was administered
				*Maintenance:* 5 mg/kg/day for 1st 10 days of life.			
Bucher [22]	Switzerland	≤32 weeks’ GA, with spontaneous respiration for ≥24 h at 48 h of age	50	*Loading:* 20 mg/kg (2 mL/kg) caffeine citrate IV at 48 h of age	*Loading & Maintenance:* Equivolume doses of IV saline 0.9%	Intermittent hypoxemia	
				*Maintenance:* 10 mg/kg (1 mL/kg) caffeine citrate IV given at 72 and 96 h of age			
Erenberg [23, 47]	USA	28–32 weeks’ GA; >24 h old; ≥6 apnea episodes within a 24-h period	82	*Loading:* 20 mg/kg (1 mL/kg) caffeine citrate IV	*Loading & Maintenance:* Equivolume matching citric salt placebo solution	Apnea	
				*Maintenance:* Caffeine citrate 5 mg/kg (0.25 ml/kg) IV/OG/NG Q24h, starting 24 h after loading dose			
Fakoor [24]	Iran	GA ≤ 32 weeks’; BW ≤ 1500 g	100	*Loading:* 20 mg/kg “venous caffeine”	No treatment	Apnea	Retrospective trial registration also states infants had to be at least 24 h of age and have “self-contained breathing” in the first 24 h of life.
				*Maintenance:* 5 mg/kg/day “venous caffeine”			
Iranpour [34]	Iran	GA ≤ 37 weeks’; BW 1250–2000 g; weight appropriate for age. Spontaneous respiration and clinical signs of respiratory distress requiring nasal CPAP	90	*Loading:* 20 mg/kg IV caffeine citrate	No treatment	Duration of positive pressure support	
				*Maintenance:* 10 mg/kg/day IV or PO (when enteral feeding) caffeine citrate, until respiratory support not required			
Liu [25]	China	GA ≤ 32 weeks’; BW < 1500 g	194	*Loading:* 20 mg/kg IV caffeine citrates within 72 h of birth	*Loading & Maintenance:* Equivolume doses of IV saline 0.9%	White matter abnormality on cerebral magnetic resonance imaging (MRI)	Study was primarily looking at cranial MRI changes, but also included outcomes relating to respiration and short-term complications of caffeine treatment
				*Maintenance:* 5 mg/kg/day IV caffeine citrate			
Murat [31]	France	GA 29–35 weeks’; ≥3 episodes of idiopathic apnea on cardiorespiratory monitoring within a 24 h period.	18	*Loading:* 20 mg/kg IM caffeine citrate (0.8 mL)	No treatment	Apnea	
				*Maintenance:* 5 mg/kg/day PO caffeine citrate			
Schmidt [33, 37, 38, 45, 48–51]	Australia, Canada, Germany Israel, The Netherlands, Sweeden, Switzerland, UK, USA	BW 500–1250 g; Day 1–10 of life; infant considered a candidate for methylxanthine therapy by clinical staff	2006	*Loading:* 20 mg/kg IV caffeine citrate:	*Loading & Maintenance:* Equivolume doses of saline 0.9%	Neurocognitive impairment (composite of death, cerebral palsy, cognitive delay, deafness or blindness) at a corrected age of 18–21 months^c^	If apnea persisted, the daily dose of caffeine could be increased to 10 mg/kg/day.
				*Maintenance:* 5 mg/kg/day IV or PO (when tolerating full enteral feeds) caffeine citrate			
Comparison 2: high-dose vs low-dose caffeine							
Kori [26]	Malaysia	GA 26–32; within 24 h peri-extubation period if ventilated	78	*Loading:* 20 mg/kg PO caffeine citrate	*Loading:* 40 mg/kg PO caffeine citrate	Apnea	
				*Maintenance:* 10 mg/kg PO caffeine citrate	*Maintenance:* 20 mg/kg PO caffeine citrate		
Mohammed [27]	Egypt	GA < 32 weeks’; AOP in first 10 days of life	120	*Loading:* 20 mg/kg IV caffeine citrate	*Loading:* 40 mg/kg IV caffeine citrate	Extubation failure in mechanically ventilated infants	
				*Maintenance:* 10 mg/kg/day IV or PO caffeine citrate, until 7 days post-extubation	*Maintenance:* 20 mg/kg/day IV/ PO caffeine citrate, until 7 days post-extubation		
Oliphant [32, 52]	New Zealand	GA 34–36 weeks’; <72 h old	132	*Loading:* Caffeine citrate 10, 20, 30 or 40 mg/kg/day PO	*Loading & Maintenance:* Equivolume doses of PO water	Intermittent hypoxemia	Trial included multiple doses and placebo arm. Data included in both comparisons (all caffeine groups vs placebo in Comparison 1; 5 & 10 mg/kg/day groups vs 15 & 20 mg/kg/day in Comparison 2).
				*Maintenance:* Caffeine citrate 5, 10, 15 or 20 mg/kg/day PO			
Scanlon [28]	England	GA < 31 weeks’; either ≥10 apnea in 8 h or ≥4 apnea in 1 h.	30	*Loading:* 25 mg/kg PO caffeine citrate	*Loading:* 50 mg/kg PO caffeine citrate (given as 2 × 25 mg/kg doses 1 h apart)	Apnea	A third group receiving theophylline was also included in the trial, but not considered in this systematic review
				*Maintenance:* 6 mg/kg/day PO caffeine citrate	*Maintenance:* 12 mg/kg/day PO caffeine citrate		
Steer [29]	Australia	GA ≤ 31 weeks’; received/were anticipated to receive ≥48 h mechanical ventilation	127	*Loading:* 30 or 60 mg/kg IV caffeine citrate	*Loading:* 6 mg/kg IV caffeine citrate	Extubation failure in mechanically ventilated infants	
				*Maintenance:* 15 or 30 mg/kg IV caffeine citrate (OG if enterally fed)	*Maintenance:* 3 mg/kg IV caffeine citrate (OG if enterally fed)		
Steer [36, 53]	Australia	Infants requiring methylxanthines for treatment of apnea of prematurity or as a part of peri-extubation management	287	*Loading:* 80 mg/kg caffeine citrate	*Loading:* 20 mg/kg caffeine citrate	Extubation failure in mechanically ventilated infants	Route of administration not specified
				*Maintenance:* 20 mg/kg caffeine citrate every 24 h, starting 24 h after loading dose	*Maintenance:* 5 mg/kg caffeine citrate every 24 h, starting 24 h after loading dose		
Zhao [30]	China	GA ≤ 32 weeks’; primary apnea	164	*Loading:* 20 mg/kg IV caffeine citrate	*Loading:* 20 mg/kg IV caffeine citrate	Apnea	
				*Maintenance:* 15 mg/kg/day IV caffeine citrate	*Maintenance:* 5 mg/kg/day IV caffeine citrate		
				*Maintenance:* 15 mg/kg/day IV caffeine citrate	*Maintenance:* 5 mg/kg/day IV caffeine citrate		

^a^All trials were randomized on a 1:1 basis (1:1:1 and 1:1:1:1:1 for the multi-arm studies Scanlon [28] and Oliphant 2022 respectively).

^b^Outcomes are for the neonatal/infant epoch unless otherwise stated.

^c^Schmidt [33] reported outcomes within the early childhood epoch at both 18–21 months and 5 years of age. In accordance with the prespecified protocol, where an outcome was measured at both time points, the latest available data (at 5 years of age) were used in meta-analysis.

**Table 2:**
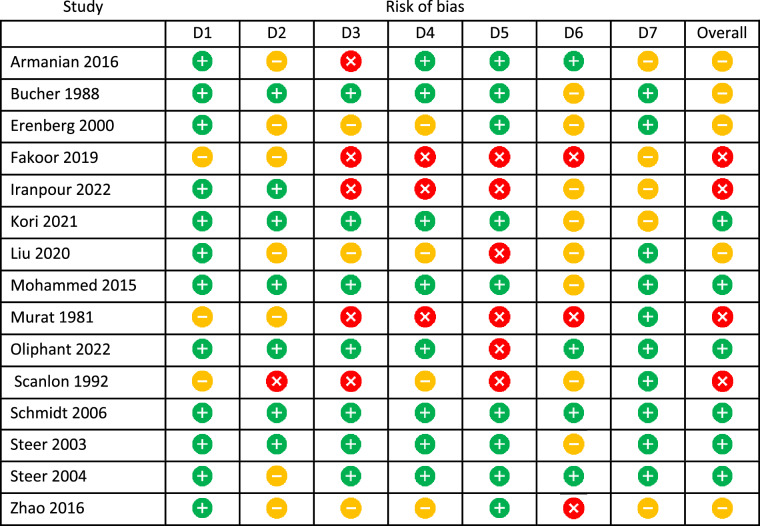
Overall risk of bias of included studies.

Key:  high,  unclear  low risk of bias for D1: Random sequence generation, D2: Allocation concealment, D3: Blinding of participants and personnel, D4: Blinding of outcome assessment, D5: Incomplete outcome data, D6: Selective reporting, D7: Other sources of bias.

#### Caffeine vs. placebo/no treatment

##### Primary outcome

Neonatal/infancy: For the primary outcome of apnea (dichotomous), evidence of very low certainty from five trials showed possible benefit from receiving caffeine compared to placebo or no treatment (risk ratio [RR] 0.59, 95% confidence interval [CI] 0.46, 0.75, 453 infants) (Table 3) [23, 24, 32, 34, 35].

**Table 3:** GRADE summary of findings for caffeine vs placebo comparison.

	Certainty Assessment					Number of patients^a^			Effect		Certainty		Importance
	Number of studies	Risk of bias	Inconsistency	Indirectness	Imprecision	Caffeine	Placebo / no treatment		Relative (95% CI)	Absolute (95% CI)			
Neonatal and infant epoch^b^													
Apnea (dichotomous outcome)	5 [23, 24, 32, 34, 35]	Very serious^c^	Serious^d^	Not serious	Serious^e^	47/271 (17.3%)	71/182 (39.0%)		RR 0.59 (0.46–0.75)	160 fewer per 1000 (from 211 fewer to 98 fewer)	⨁◯◯◯ Very low		Critical
Apnea (continuous outcome)	2 [25, 31]	Very serious^f^	Very serious^g^	Not serious	Serious^e^	1.8 events / day (*N* = 89)	1.9 events / day (*N* = 86)		-	MD 0.7 lower (1.1 lower to 0.2 lower)	⨁◯◯◯ Very low		Critical
Intraventricular hemorrhage	3 [24, 34, 35]	Very serious^h^	Not serious	Not serious	Very serious^i^	9/121 (7.4%)	5/121 (4.1%)		RR 1.80 (0.64–5.03)	33 more per 1000 (from 15 fewer to 167 more)	⨁◯◯◯ Very low		Critical
Death before primary hospital discharge	7 [23–25, 32–35]	Serious^j^	Not serious	Not serious	Serious^e^	70/1370 (5.1%)	69/1275 (5.4%)		RR 1.00 (0.73–1.38)	0 fewer per 1000 (from 15 fewer to 21 more)	⨁⨁◯◯ Low		Critical
Intermittent hypoxemia	2 [22, 32]	Not serious	Not serious	Serious^k^	Serious^e^	1.0 events / day (*N* = 110)	0.7 events / day (*N* = 47)		-	MD 0.3 higher (0.2 higher to 0.4 higher)	⨁⨁◯◯ Low		Important
Bronchopulmonary dysplasia	3 [33–35]	Serious^l^	Not serious	Not serious	Not serious	363/1034 (35.1%)	466/1025 (45.5%)		RR 0.77 (0.69–0.86)	105 fewer per 1000 (from 141 fewer to 64 fewer)	⨁⨁⨁◯ Moderate		Important
Duration of mechanical ventilation	2 [24, 25]	Very Serious^m^	Serious^n^	Not serious	Serious^e^	5.0 days (*N* = 130)	4.3 days (*N* = 130)		-	MD 1.2 lower (2.5 lower to 0.1 higher)	⨁◯◯◯ Very low		Important
Duration of positive pressure support	2 [24, 34]	Very Serious^o^	Serious^d^	Not serious	Serious^e^	2.6 days (*N* = 95)	3.0 days (*N* = 95)		-	MD 0.2 lower (1.0 lower to 0.6 higher)	⨁◯◯◯ Very low		Important
Tachycardia	5 [22, 25, 32, 34, 35]	Serious^p^	Not serious	Not serious	Very serious^i^	21/262 (8.0%)	14/198 (7.1%)		RR 1.50 (0.81–2.79)	35 more per 1000 (from 13 fewer to 127 more)	⨁◯◯◯ Very low		Important
Patent ductus arteriosus	4 [24, 33–35]	Serious^q^	Not serious	Not serious	Not serious	352/1122 (31.4%)	525/1120 (46.9%)		RR 0.67 (0.60–0.74)	155 fewer per 1000 (from 188 fewer to 122 fewer)	⨁⨁⨁◯ Moderate		Important
Growth velocity – weight gain	1 [32]	Not serious	Serious^r^	Not serious	Very serious^i^	6.2 g/kg/d (*N* = 77)	8.8 g/kg/d (*N* = 20)		-	MD 2.6 lower (4.2 lower to 1.0 lower)	⨁◯◯◯ Very low		Important
Growth velocity – linear growth	1 [32]	Not serious	Serious^r^	Not serious	Very serious^i^	0.8 cm/week (*N* = 77)	0.7 cm/week (*N* = 20)		-	MD 0.1 higher (0.2 lower to 0.4 higher)	⨁◯◯◯ Very low		Important
Growth velocity – head circumference	1 [32]	Not serious	Serious^r^	Not serious	Very Serious^i^	0.5 cm/week (*N* = 77)	0.6 cm/week (*N* = 20)		-	MD 0.1 lower (0.2 lower to 0.0 higher)	⨁◯◯◯ Very low		Important
Time to establish full enteral feeds	1 [34]	Very serious^s^	Not serious	Not serious	Serious^e^	154 h (*N* = 45)	171 h (*N* = 45)		-	MD 17 fewer hours (43 fewer to 8 more)	⨁◯◯◯ Very low		Less important
Early childhood epoch^t^													
Neurocognitive impairment	1 [37]	Not serious	Serious^r^	Not serious	Serious^e^	38/768 (4.9%)	38/750 (5.1%)		RR 0.98 (0.63–1.51)	1 fewer per 1000 (from 19 fewer to 26 more)	⨁⨁◯◯ Low		Critical
Death	1 [37]	Not serious	Serious^r^	Not serious	Serious^e^	59/867 (6.8%)	58/837 (6.9%)		RR 0.98 (0.69–1.39)	1 fewer per 1000 (from 21 fewer to 27 more)	⨁⨁◯◯ Low		Critical
Survival without neurosensory impairment	1 [37]	Not serious	Serious^r^	Not serious	Not serious	657/833 (78.9%)	607/807 (75.2%)		RR 1.05 (0.99–1.11)	38 more per 1000 (from 8 fewer to 83 more)	⨁⨁⨁◯ Moderate		Critical
Motor Impairment	1 [37]	Not serious	Serious^r^	Not serious	Serious^e^	13/803 (1.6%)	21/773 (2.7%)		RR 0.60 (0.30–1.18)	11 fewer per 1000 (from 19 fewer to 5 more)	⨁⨁◯◯ Low		Critical
Cerebral palsy	1 [37]	Not serious	Serious^r^	Not serious	Serious^e^	40/909 (4.4%)	66/901 (7.3%)		RR 0.60 (0.41–0.88)	29 fewer per 1000 (from 43 fewer to 9 fewer)	⨁⨁◯◯ Low		Critical
Hearing impairment	1 [37]	Not serious	Serious^r^	Not serious	Serious^e^	22/798 (2.8%)	25/773 (3.2%)		RR 0.85 (0.48–1.50)	5 fewer per 1,000 (from 17 fewer to 16 more)	⨁⨁◯◯ Low		Critical
Visual Impairment	1 [37]	Not serious	Serious^r^	Not serious	Serious^e^	7/792 (0.9%)	7/763 (0.9%)		RR 0.96 (0.34–2.73)	0 fewer per 1000 (from 6 fewer to 16 more)	⨁⨁◯◯ Low		Critical
Emotional-behavioral difficulties	1 [37]	Not serious	Serious^r^	Not serious	Serious^e^	42/773 (5.4%)	53/748 (7.1%)		RR 0.77 (0.52–1.14)	16 fewer per 1000 (from 34 fewer to 10 more)	⨁⨁◯◯ Low		Important
Growth - Weight	1 [37]	Not serious	Serious^r^	Not serious	Not serious	−0.19 Z-score (*N* = 798)	−0.16 Z-score (*N* = 763)		-	MD 0.03 lower (0.08 lower to 0.02 higher)	⨁⨁⨁◯ Moderate		Important
Growth – Height	1 [37]	Not serious	Serious^r^	Not serious	Not serious	−0.04 Z-score (*N* = 793)	−0.04 Z-score (*N* = 759)		-	MD 0.03 lower (0.08 lower to 0.02 higher)	⨁⨁⨁◯ Moderate		Important
Middle childhood epoch^u^													
Neurocognitive impairment	1 [38]	Not serious	Serious^r^	Not serious	Not serious	145/457 (31.7%)		174/463 (37.6%)	RR 0.84 (0.71–1.01)	60 fewer per 1000 (from 109 fewer to 4 more)		⨁⨁⨁◯ Moderate	Critical
Motor Impairment	1 [38]	Not serious	Serious^r^	Not serious	Not serious	90/457 (19.7%)		130/473 (27.5%)	RR 0.72 (0.57–0.91)	77 fewer per 1000 (from 118 fewer to 25 fewer)		⨁⨁⨁◯ Moderate	Critical
Cerebral palsy	1 [38]	Not serious	Serious^r^	Not serious	Serious^e^	21/484 (4.3%)		29/484 (6.0%)	RR 0.72 (0.42–1.25)	17 fewer per 1000 (from 35 fewer to 15 more)		⨁⨁◯◯ Low	Critical
Hearing impairment	1 [38]	Not serious	Serious^r^	Not serious	Serious^e^	16/484 (3.3%)		13/484 (2.7%)	RR 1.23 (0.60–2.53)	6 more per 1000 (from 11 fewer to 41 more)		⨁⨁◯◯ Low	Critical
Visual Impairment	1 [38]	Not serious	Serious^r^	Not serious	Very serious^i^	4/484 (0.8%)		1/484 (0.2%)	RR 4.00 (0.45–35.66)	6 more per 1000 (from 1 fewer to 72 more)		⨁◯◯◯ Very low	Critical
Emotional-behavioral difficulties	1 [38]	Not serious	Serious^r^	Not serious	Very serious^i^	52/476 (10.9%)		40/481 (8.3%)	RR 1.31 (0.89–1.94)	26 more per 1000 (from 9 fewer to 78 more)		⨁◯◯◯ Very low	Important
Asthma/Wheeze	1 [38]	Not serious	Serious^r^	Not serious	Very serious^i^	10/88 (11.4%)		17/80 (21.3%)	RR 0.53 (0.26–1.10)	100 fewer per 1000 (from 157 fewer to 21 more)		⨁◯◯◯ Very low	Important
Growth - Weight	1 [38]	Not serious	Serious^r^	Not serious	Not serious	−0.18 Z-score (*N* = 474)		−0.10 Z-score (*N* = 479)	-	MD 0.08 lower (0.24 lower to 0.08 higher)		⨁⨁⨁◯ Moderate	Important
Growth – Height	1 [38]	Not serious	Serious^r^	Not serious	Not serious	−0.20 Z-score (*N* = 474)		−0.21 Z-score (*N* = 478)	-	MD 0.01 higher (0.13 lower to 0.15 higher)		⨁⨁⨁◯ Moderate	Important

^a^For continuous outcomes values represent weighted mean.

^b^In the neonatal and infant epoch, the critical outcomes of death before one year of age, neurocognitive impairment, survival without neurosensory impairment and cerebral palsy and the important outcome of retinopathy of prematurity were not reported by any included studies.

^c^Two included studies (Fakoor [24], Iranpour [34] were judged to have high overall risk of bias; two (Armanian [35], Erenberg [23]) were judged to have some concerns overall and one (Oliphant 2022) was judged to have a low overall risk of bias for this outcome.

^d^*I*^2^ = 78%.

^e^OIS criteria not met (total population less than optimal information size [OIS] resulting in downgrading by one step).

^f^One included study (Liu [25]) was judged to have high overall risk of bias for this outcome, and the other (Murat 1981) was judged to have some concerns overall for this outcome.

^g^*I*^2^ = 97%.

^h^Two included studies (Fakoor [24] & Iranpour [34]) were judged to have high overall risk of bias for this outcome and one (Armanian [35]) was judged to have some concerns overall for this outcome.

^i^OIS criteria not met (total population less than half of OIS, resulting in downgrading by two steps).

^j^Two included studies (Fakoor [24] & Iranpour [34]) were judged to have high overall risk of bias for this outcome; three (Armanian [35], Erenberg [23] & Liu [25]) were judged to have some concerns overall for this outcome; and two (Oliphant 2022 & Schmidt [33]) were  judged to have low risk of bias overall for this outcome.

^k^Patient populations of the two included studies were substantially different: Bucher [22] included infants under 32 weeks’ gestation (mean 30.3 weeks) while Oliphant 2022 included infants 34–36 weeks’ gestation. Intermittent hypoxemia is known to vary by gestational age.

^l^One included study (Iranpour 2022) was judged to have high overall risk of bias for this outcome; one (Armanian [35]) was judged to have some concerns overall for this outcome; and one (Schmidt [33]) was judged to have low risk of bias overall for this outcome.

^m^One included study (Fakoor [24]) was judged to have high overall risk of bias for this outcome; and one (Liu [25]) was judged to have some concerns overall for this outcome.

^n^*I*^2^ = 43%.

^o^Both included studies (Fakoor [24] & Iranpour [34]) were judged to have high overall risk of bias for this outcome.

^p^One included study (Iranpour [34]) was judged to have high overall risk of bias for this outcome; three (Armanian [35], Bucher [22] & Liu [25]) were judged to have some concerns overall for this outcome; and one (Oliphant 2022) was judged to have low risk of bias overall for this outcome.

^q^Two included studies (Fakoor [24] & Iranpour [34]) were judged to have high overall risk of bias for this outcome; one (Armanian [35]) was judged to have some concerns overall for this outcome; and one (Schmidt [33]) was judged to have low risk of bias overall for this outcome.

^r^Results from a single study only.

^s^The only included study (Iranpour [34]) was judged to have high overall risk of bias for this outcome.

^t^In the early childhood epoch, the important outcome of asthma / wheeze was not reported by any included studies.

^u^In the middle childhood epoch, the critical outcomes of death before one year of age and survival without neurosensory impairment were not reported by any included studies.

**Fig. 2:**
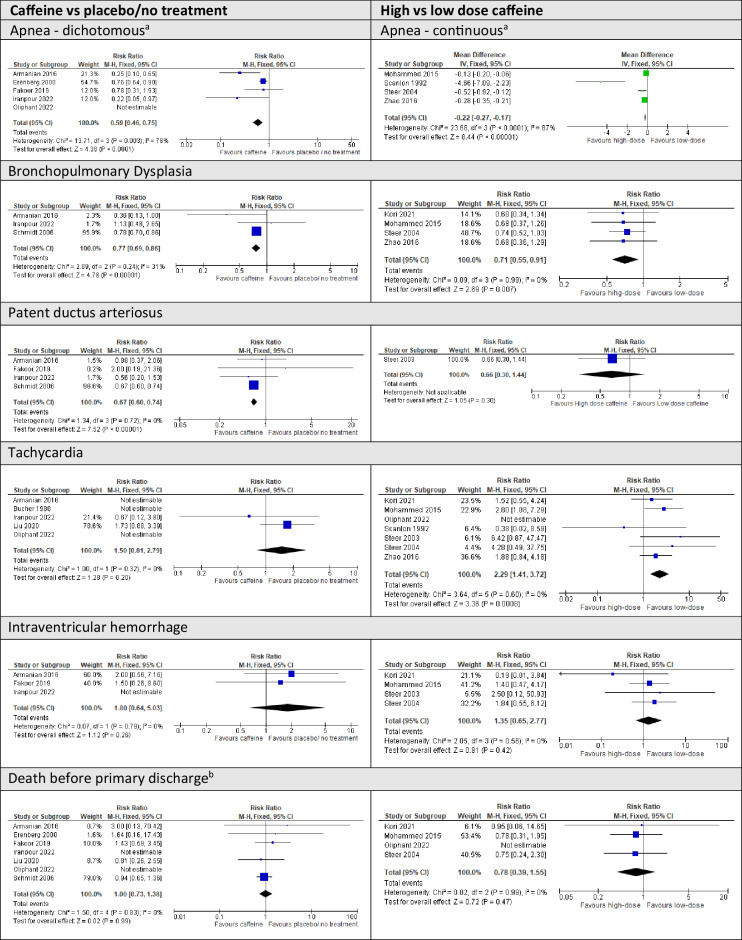
Forest plots of the neonatal/infant primary outcome, and critical and selected important secondary outcomes. ^a^Apnea results are presented as a dichotomous measure (for caffeine vs placebo comparison) or a continuous measure (for high vs low-dose comparison), based on how apnea was measured in the majority of studies in each comparison. The forest plot for the alternate measure for each comparison is presented in Fig. 3. ^b^Death before one year of age was also considered a critical outcome, but only 1 study reported this measure (in the low vs. high-dose comparison). This data is included in Fig. 3, with other secondary outcomes.

There was statistical heterogeneity (*I*^2^ = 78%) among trials, although the direction of effect consistently favored caffeine (Fig. 2). In sensitivity analysis, exclusion of two trials at high risk of bias [24, 34], did not substantially alter the results (RR 0.62, 95% CI 0.50, 0.77, three trials, 263 infants).

Early childhood: For the primary outcome of neurocognitive impairment, evidence of low certainty from one trial could not exclude clinical benefit or harm from receiving caffeine compared to placebo (RR 0.98, 95% 0.63, 1.51, 1518 children) (Table 3) [37].

Middle childhood: For the primary outcome of neurocognitive impairment, evidence of moderate certainty showed possible benefit from receiving caffeine compared to placebo (RR 0.84, 95% 0.71, 1.01, 1 trial, 920 children) (Table 3) [38].

There were no data for the primary outcome of neurocognitive impairment in adolescence.

##### Secondary outcomes

**Fig. 3:**
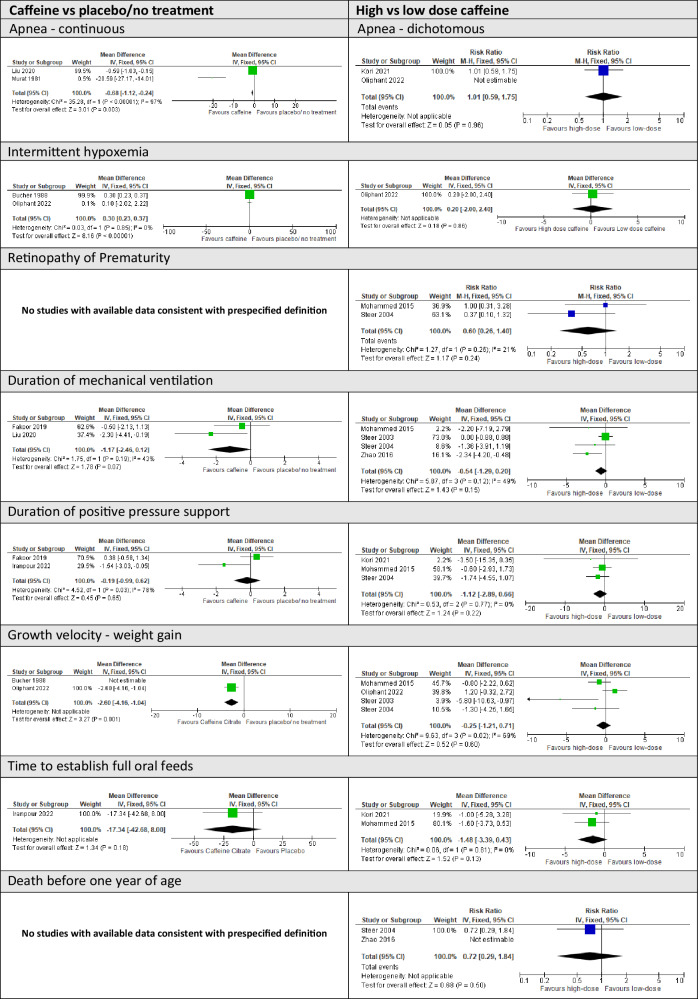
Forest plots of additional neonatal/infant secondary outcomes.

Moderate certainty evidence indicated probable clinical benefit of receiving caffeine compared to placebo or no treatment for BPD (RR 0.77, 95% CI 0.69, 0.86, three trials, 2059 infants, *I*^2^ = 31%) and patent ductus arteriosus (RR 0.67, 95% CI 0.60, 0.74, four trials, 2242 infants, *I*^2^ = 0%)(Table 3), and motor impairment in middle childhood (RR 0.72 95% CI 0.57, 0.91, one trial, 930 infants) (Table 3). Caffeine therapy may reduce neurocognitive impairment and cerebral palsy (Table 3). It is possible that caffeine reduces weight gain velocity after birth, but it does not appear to affect body size in childhood (Table 3). The evidence was too uncertain to determine the effect of caffeine on intermittent hypoxemia, respiratory support, feeding, other major neonatal morbidities, death, other developmental outcomes in childhood, and asthma/wheeze (Table 3; Fig. 3).

##### Secondary analysis

There were insufficient data to undertake the planned subgroup analyses.

#### High-dose vs. low-dose caffeine

##### Primary outcome

Neonatal/infancy: For the primary outcome of apnea (continuous), evidence of very low certainty from four trials showed possible benefit from receiving high-dose caffeine compared to low-dose caffeine, although the effect size was small (mean difference [MD] −0.2, 95% CI −0.3, −0.2, 560 infants) (Table 4) [27, 28, 30, 36]. There was statistical heterogeneity (*I*^2^ = 87%) among trials, although the direction of effect consistently favored high-dose caffeine (Fig. 2). In sensitivity analysis, exclusion of one trial at high risk of bias [28], did not alter the results (MD −0.2, 95% CI −0.3, −0.2, 530 infants, *I*^2^ = 81%).

**Table 4:** GRADE summary of findings for low vs high-dose caffeine comparison.

Outcome	Certainty Assessment							Number of patients^a^		Effect		Certainty	Importance
	Number of studies	Risk of bias		Inconsistency	Indirectness	Imprecision		High-dose caffeine	Low-dose caffeine	Relative (95% CI)	Absolute (95% CI)		
Neonatal and infant epoch^b^													
Apnea (dichotomous outcome)	2 [26, 32]	Not serious		Serious^c^	Not serious	Very serious^d^		16/94 (17.0%)	15/89 (16.9%)	RR 1.01 (0.59–1.75)	2 more per 1000 (from 69 fewer to 126 more)	⨁◯◯◯ Very low	Critical
Apnea (continuous outcome)	4 [27, 28, 30, 36]	Serious^e^		Very serious^f^	Not serious	Not serious		0.1 events / day (*N* = 276)	0.5 events / day (*N* = 284)	-	MD 0.2 lower (0.3 lower to 0.2 lower)	⨁◯◯◯ Very low	Critical
Intraventricular hemorrhage	4 [26, 27, 29, 36]	Not serious		Not serious	Not serious	Very serious^d^		16/305 (5.2%)	11/266 (4.1%)	RR 1.35 (0.65–2.77)	14 more per 1000 (from 14 fewer to 73 more)	⨁⨁◯◯ Low	Critical
Death before primary hospital discharge	5 [26, 27, 32, 36]	Not serious		Not serious	Not serious	Very serious^d^		21/356 (5.9%)	28/357 (7.8%)	RR 0.76 (0.44–1.30)	19 fewer per 1000 (from 44 fewer to 24 more)	⨁⨁◯◯ Low	Critical
Death before one year of age	1 [36]	Serious^g^		Serious^h^	Not serious	Very Serious^d^		7/116 (6.0%)	10/120 (8.3%)	RR 0.72 (0.29–1.84)	23 fewer per 1000 (from 59 fewer to 70 more)	⨁◯◯◯ Very low	Critical
Intermittent hypoxemia	1 [32]	Not serious		Serious^h^	Not serious	Very serious^d^		4.8 events/h (*N* = 41)	4.6 events/h (*N* = 44)	-	MD 0.2 higher (2.0 lower to 2.4 higher)	⨁◯◯◯ Very low	Important
Bronchopulmonary dysplasia	4 [26, 27, 30, 36]	Not serious		Not serious	Not serious	Serious^i^		71/289 (24.6%)	104/297 (35.0%)	RR 0.71 (0.55–0.91)	102 fewer per 1000 (from 158 fewer to 32 fewer)	⨁⨁⨁◯ Moderate	Important
Duration of mechanical ventilation	4 [27, 29, 30, 36]	Serious^j^		Serious^k^	Not serious	Not serious		2.8 days (*N* = 347)	1.7 days (*N* = 310)	-	MD 0.54 lower (1.3 lower to 0.2 higher)	⨁⨁◯◯ Low	Important
Duration of positive pressure support	3 [26, 27, 36]	Not serious		Not serious	Not serious	Very serious^d^		3.8 days (*N* = 220)	4.3 days (*N* = 224)	-	MD 1.1 lower (2.9 lower to 0.7 higher)	⨁⨁◯◯ Low	Important
Tachycardia	7 [26–30, 32, 36]	Serious^l^		Not serious	Not serious	Very serious^d^		54/435 (12.4%)	21/404 (5.2%)	RR 2.29 (1.41–3.72)	67 more per 1000 (from 21 more to 141 more)	⨁◯◯◯ Very low	Important
Patent ductus arteriosus	1 [29]	Not serious		Serious^h^	Not serious	Very serious^d^		12/85 (14.1%)	9/42 (21.4%)	RR 0.66 (0.30–1.44)	73 fewer per 1000 (from 150 fewer to 94 more)	⨁◯◯◯ Very low	Important
Retinopathy of Prematurity	2 [27, 36]	Not serious		Not serious	Not serious	Very serious^d^		8/153 (5.2%)	14/163 (8.6%)	RR 0.60 (0.26–1.40)	34 fewer per 1000 (from 64 fewer to 34 more)	⨁⨁◯◯ Low	Important
Growth velocity – weight gain	4 [27, 29, 32, 36]	Not serious		Very serious^m^	Not serious	Not serious		8.6 g/kg/d (*N* = 302)	9.5 g/kg/d (*N* = 268)	-	MD 0.3 lower (1.2 lower to 0.7 higher)	⨁⨁◯◯ Low	Important
Growth velocity – linear growth	1 [32]	Not serious		Serious^h^	Not serious	Very serious^d^		0.8 cm/week (N = 37)	0.7 cm/week (N = 40)	-	MD 0.1 higher (0.1 lower to 0.3 higher)	⨁◯◯◯ Very low	Important
Growth velocity – head circumference	1 [32]	Not serious		Serious^h^	Not serious	Very serious^d^		0.5 cm/week (*N* = 37)	0.5 cm/week (*N* = 40)	-	MD 0.0 (0.1 lower to 0.1 higher)	⨁◯◯◯ Very low	Important
Time to full enteral feeds	2 [26, 27]	Not serious		Not serious	Not serious	Serious^i^		12.3 days (*N* = 100)	11.5 days (*N* = 98)	-	MD 1.5 fewer days (3.4 fewer to 0.4 more)	⨁⨁⨁◯ Moderate	Less important
Early childhood epoch^n^													
Survival without neurosensory impairment	1 [36]	Not serious		Serious^h^	Not serious	Serious^i^		95/120 (79.2%)	100/116 (86.2%)	RR 0.92 (0.82–1.03)	69 fewer per 1000 (from 155 fewer to 26 more)	⨁⨁◯◯ Low	Critical

^a^For continuous outcomes values represent weighted mean.

^b^In the neonatal and infant epoch, the critical outcomes of neurocognitive impairment, survival without neurosensory impairment and cerebral palsy were not reported by any included studies.

^c^Although 2 studies reported apnea as an outcome, the apnea outcome did not occur in any participants in Oliphant 2022, and hence only a single study (Kori [26]) contributed data to this analysis.

^d^OIS criteria not met (total population less than half of OIS, resulting in downgrading by two steps).

^e^One included study (Scanlon [28]) was judged to have high overall risk of bias for this outcome, two (Steer [36] & Zhao [30]) were judged to have some concerns overall for this outcome and one (Mohammed [27]) was judged to have a low overall risk of bias for this outcome.

^f^*I*^2^ = 87%.

^g^Data are from a single study (Steer [36]) with a high risk of incomplete outcome data.

^h^Results from a single study only.

^i^OIS criteria not met (total population less than OIS, resulting in downgrading by one step).

^j^Two included studies (Steer [36] & Zhao [30]) were judged to have some concerns overall for this outcome and two (Mohammed [27], Steer [29]) were judged to have a low overall risk of bias for this outcome.

^k^*I*^2^ = 49%.

^l^One included study (Scanlon [28]) was judged to have high overall risk of bias for this outcome; two (Steer [36] & Zhao [30]) were judged to have some concerns overall for this outcome; and the remaining four studies (Kori [26], Mohammed [27], Oliphant 2022 & Steer [29]) were judged to have a low overall risk of bias for this outcome.

^m^*I*^2^ = 69%.

^n^In the early childhood epoch, the critical outcomes of death, neurocognitive impairment, motor impairment, cerebral palsy, hearing impairment and visual impairment, and the important outcomes of emotional-behavioral difficulties, asthma/wheeze, growth – height and growth – height were not reported by any included studies.

Other epochs: No trials of high-dose vs. low-dose caffeine reported on neurocognitive impairment.

##### Secondary outcomes

Moderate certainty evidence from four trials showed probable benefit for BPD with high-dose vs. low-dose caffeine (RR 0.71 95% CI 0.55, 0.91, 586 infants, *I*^2^ = 0%)(Table 4). Evidence of very low certainty from seven trials suggested that high-dose vs. low-dose caffeine may increase the rate of tachycardia (RR 2.29 95%CI 1.41, 3.72, 839 infants, *I*^2^ = 0%)(Table 4). The evidence was too uncertain to determine the effect of high-dose vs. low-dose caffeine on other neonatal outcomes (Table 4; Fig. 3). For the critical outcome of survival without neurosensory impairment in early childhood, low certainty evidence from one trial meant that a benefit of high-dose vs. low-dose caffeine could not be excluded (RR 0.92 95%CI 0.82, 1.03, 236 children) (Table 4).

##### Secondary analysis

There were insufficient data to undertake the planned subgroup analyses.

## Discussion

Currently, there is no high-certainty evidence for use of caffeine in preterm neonates for any critical or important outcomes from birth to adolescence. However, in very preterm neonates, caffeine therapy probably reduces the rate of BPD and PDA; possibly increases survival without neurosensory impairment in early childhood and reduces cerebral palsy; and probably reduces the rate of neurocognitive impairment and motor impairment in middle childhood. Although traditionally given for apnea of prematurity, the evidence supporting this benefit of caffeine was of very low certainty, given the considerable heterogeneity in contributing studies, RoB inherent in these studies and the relatively small number of infants for whom data are available.

In general, evidence for the relative effectiveness of high vs. low-dose caffeine is even less certain, but moderate certainty evidence indicates higher doses probably reduce the rate of BPD more than lower ones, and very low certainty evidence suggests higher doses may cause more tachycardia.

Quantifying the effect of caffeine on longer-term outcomes is limited by the available studies, with only two trials presenting any outcome data beyond the neonatal/infancy period (one in each comparison) and only one of those reporting significant follow-up assessments and results. As a result, meta-analysis was not possible in epochs beyond neonatal/infancy, and the certainty of the findings is limited. No information was available comparing the effects of high and low-dose caffeine on neurodevelopmental outcomes.

This review provides a current and comprehensive summary of the available literature on the use of caffeine in preterm infants and included 15 RCTs covering 3530 premature infants. In contrast to previous systematic reviews, we included all studies enrolling preterm infants (<37 weeks’ PMA), rather than limiting the population to infants born at earlier gestational ages [39–41]. This was because moderate and late preterm infants may experience apnea of prematurity [2] and are known to have episodes of intermittent hypoxemia [3], and so may also benefit from caffeine therapy, though the evidence in this are remains uncertain. Previous systematic reviews have addressed a single question (either caffeine vs. placebo, or high vs. low-dose regimens), rather than considering both together as in this review, and have often included trials of other methylxanthines which are no longer routinely used in addition to caffeine. Furthermore, these older systematic reviews did not apply the explicit and comprehensive GRADE criteria to the assessment of the quality of the evidence, and so have perhaps overstated the certainty of the evidence underlying their recommendations [10, 42]. Recently published Cochrane reviews present GRADE analysis for only a subset of outcomes [41, 43], whereas in this review GRADE analysis was performed for all outcomes with available data.

The Cochrane Neonatal Group have recently published reviews of caffeine dosing regimens in preterm infants [41] and of methylxanthines vs placebo / no treatment [43]. However, this later Cochrane review includes a substantial number of trials that used other methylxanthines (aminophylline and theophylline) no longer routinely used in clinical practice and does not include some of the more recent trials of caffeine [24, 32, 34] included in this review. Both the Cochrane and other reviews of caffeine low-dose vs high-dose caffeine therapy have concluded that higher doses of caffeine are [44] or may be [40, 41] more effective in reducing the occurrence of extubation failure. Analysis of the evidence for the important outcome of BPD has resulted in different conclusions in different reviews; either that higher doses reduce the rate of BPD compared with lower doses [39, 41, 44] or that higher doses do not alter the rate of BPD [40]. In contrast to previously published reviews [39, 41, 44], we pre-defined high (>10 mg·kg^−1^day^−1^ caffeine citrate equivalent) and low doses (≤10 mg·kg^−1^day^−1^) of caffeine on the basis of maintenance dose, avoiding cross-over of doses included in the comparison groups and hence producing a more meaningful comparison. This may explain the differences in findings, as some other reviews have included trials where the only difference in dose was in the loading dose [39, 40], or where both doses used would be considered low doses in current clinical practice [39]. We also included all trials where infants received caffeine, regardless of indication, as we wished to include apnea given for the prevention of neurodevelopmental impairment, as well as solely for the prevention or treatment of apnea, or to assist in extubation.

As a systematic review, the robustness of the conclusions is limited by the quality and quantity of the included studies. The caffeine vs. placebo comparison identified and included a number of recent studies that have not previously been included in published meta-analysis [24, 30, 34], but some of these studies have domains with high risk of bias and there was a high degree of heterogeneity between studies, limiting the quality of the evidence. Furthermore, this comparison is dominated by a single study, which contributed over 2000 infants of the 2592 participants identified [45]. We had planned to undertake subgroup analysis to assess the effectiveness of caffeine based on the indication for use (prophylaxis, treatment of apnea or for extubation, late hypoxemia or established lung disease) and by gestation (extremely, very, moderately or late preterm) but were unable to undertake these analyses due to the lack of data broken down by these variables in the identified studies. The lack of data on the effectiveness of caffeine in these different subgroups remains an important evidence gap, and further research is needed to inform evidence-based decision-making in clinical care.

While caffeine is widely used in neonatal units, the evidence remains uncertain, and other reviews on the topic have called for further clinical trials in this area [40, 44, 46]. We join previous authors in this call for further research, and this systematic review indicates the evidence gaps where more information is required to guide clinical practice. In particular, there is a lack of data on long-term outcomes following different doses of caffeine in the neonatal period, and longer-term follow-up of infants in recent trials should be conducted to address this evidence gap. This is particularly important given the indications in this and other [39, 40, 44] meta-analyses that higher doses may be more effective in improving short-term outcomes, as use of higher doses in clinical practice should be preceded by evidence of the long-term safety of such doses. In addition to dose, more information is required on how the indication for treatment, infant gestation, duration of treatment/stopping and timing of initiation and discontinuation influence outcomes. Whether caffeine should be used during mechanical ventilation should also be ascertained, and if the dose should be decreased with tachycardia or increased with gestational age.

## Conclusion

Caffeine administered to preterm infants probably reduces BPD, PDA, and motor impairment, with higher doses probably conferring additional benefit in reducing BPD but possibly increasing the occurrence of tachycardia. However, most of the current evidence is of low certainty, and establishing the optimal dose requires more research, including long-term outcome assessment.

## Data Availability

Data used in this review was sourced from published trials. Derived data that support the findings of this study are available from the corresponding author upon reasonable request.

## References

[1] Moster D, Lie RT, Markestad T (2008). Long-term medical and social consequences of preterm birth. N Engl J Med.

[2] Henderson-Smart DJ (1981). The effect of gestational age on the incidence and duration of recurrent apnoea in newborn babies. Aust Paediatr J.

[3] Williams LZJ, McNamara D, Alsweiler JM (2018). Intermittent hypoxemia in infants born late preterm: a prospective cohort observational study. J Pediatr.

[4] Janvier A, Khairy M, Kokkotis A, Cormier C, Messmer D, Barrington K (2004). Apnea is associated with neurodevelopmental impairment in very low birth weight infants. J Perinatol.

[5] Poets CF, Roberts RS, Schmidt B, Whyte RK, Asztalos EV, Bader D (2015). Association between intermittent hypoxemia or bradycardia and late death or disability in extremely preterm infants. JAMA.

[6] Woythaler M, McCormick MC, Smith VC (2011). Late preterm infants have worse 24-month neurodevelopmental outcomes than term infants. Pediatrics.

[7] Muehlbacher T, Gaertner VD, Bassler D (2020). History of caffeine use in neonatal medicine and the role of the CAP trial. Semin Fetal Neonatal Med.

[8] Aranda JV, Gorman W, Bergsteinsson H, Gunn T (1977). Efficacy of caffeine in treatment of apnea in the low-birth-weight infant. J Pediatr.

[9] Yuan Y, Yang Y, Lei X, Dong W (2022). Caffeine and bronchopulmonary dysplasia: Clinical benefits and the mechanisms involved. Pediatr Pulmonol.

[10] Henderson-Smart DJ, De Paoli AG. Methylxanthine treatment for apnoea in preterm infants. Cochrane Database Syst Rev. 2010. 10.1002/14651858.CD000140.pub2. 10.1002/14651858.CD000140.pub2 PMC11751766 21154343

[11] Schoen K, Yu T, Stockmann C, Spigarelli MG, Sherwin CMT (2014). Use of methylxanthine therapies for the treatment and prevention of apnea of prematurity. Pediatr Drugs.

[12] Erickson G, Dobson N, Hunt C (2021). Immature control of breathing and apnea of prematurity: the known and unknown. J Perinatol.

[13] Thomas J, Chandler J, Cumpston M, Li T, Page M, Welch V (eds.). Cochrane handbook for systematic reviews of interventions. 6.3. Cochrane; 2022. https://training.cochrane.org/handbook/archive/v6.3.

[14] Page MJ, McKenzie JE, Bossuyt PM, Boutron I, Hoffmann TC, Mulrow CD (2021). The PRISMA 2020 statement: an updated guideline for reporting systematic reviews. Br Med J.

[15] Schünemann H, Brożek J, Guyatt G, Oxman A (eds.). GRADE handbook. The GRADE Working Group; 2013. https://gdt.gradepro.org/app/handbook/handbook.html.

[16] American Academy of Paediatrics. (2003). Policy statement: apnea, sudden infant death syndrome, and home monitoring. Pediatrics.

[17] Garner A, Ben-Sira I, Deutman A, Fledelius H, Flynn J, Gole G (1984). An international classification of retinopathy of prematurity. Pediatrics.

[18] Papile LA, Burstein J, Burstein R, Koffler H (1978). Incidence and evolution of subependymal and intraventricular hemorrhage: a study of infants with birth weights less than 1,500 gm. J Pediatr.

[19] Higgins J, Altman D, Gøtzsche P, Jüni P, Moher D, Oxman A (2011). The Cochrane Collaboration’s tool for assessing risk of bias in randomised trials. BMJ.

[20] Wan X, Wang W, Liu J, Tong T (2014). Estimating the sample mean and standard deviation from the sample size, median, range and/or interquartile range. BMC Med Res Methodol.

[21] Guyatt GH, Oxman AD, Kunz R, Brozek J, Alonso-Coello P, Rind D (2011). GRADE guideline 6. Rating the quality of evidence—imprecisions. J Clin Epidemiol.

[22] Bucher HU, Duc G (1988). Does caffeine prevent hypoxaemic episodes in premature infants? A randomized controlled trial. Eur J Pediatr.

[23] Erenberg A, Leff RD, Haack DG, Mosdell KW, Hicks GM, Wynne BA (2000). Caffeine citrate for the treatment of apnea of prematurity: a double-blind, placebo-controlled study. Pharmacotherapy.

[24] Fakoor Z, Makooie AA, Joudi Z, Asl RG (2019). The effect of venous caffeine on the prevention of apnea of prematurity in the very preterm infants in the neonatal intensive care unit of Shahid Motahhari Hospital, Urmia, during a year. J Adv Pharm Technol Res.

[25] Liu S, Zhang X, Liu Y, Yuan X, Yang L, Zhang R (2020). Early application of caffeine improves white matter development in very preterm infants. Respir Physiol Neurobiol.

[26] Kori AMM, Van Rostenberghe H, Ibrahim NR, Yaacob NM, Nasir A. A randomized controlled trial comparing two doses of caffeine for apnoea in prematurity. Int J Environ Res Public Health. 2021;18. 10.3390/IJERPH18094509. 10.3390/ijerph18094509 PMC8123071 33922783

[27] Mohammed S, Nour I, Shabaan AE, Shouman B, Abdel-Hady H, Nasef N (2015). High versus low-dose caffeine for apnea of prematurity: a randomized controlled trial. Eur J Pediatr.

[28] Scanlon JEM, Chin KC, Morgan MEI, Durbin GM, Hale KA, Brown SS (1992). Caffeine or theophylline for neonatal apnoea?. Arch Dis Child.

[29] Steer P, Flenady VJ, Shearman A, Lee TC, Tudehope DI, Charles BG (2003). Periextubation caffeine in preterm neonates: a randomized dose response trial. J Paediatr Child Health.

[30] Zhao Y, Tian X, Liu G (2016). Clinical effectiveness of different doses of caffeine for primary apnea in preterm infants [Article in Chinese]. Zhonghua Er Ke Za Zhi.

[31] Murat I, Moriette G, Blin MC, Couehard M, Flouvat B, De Gamarra E (1981). The efficacy of caffeine in the treatment of recurrent idiopathic apnea in premature infants. J Pediatr.

[32] Oliphant EA, McKinlay CJD, McNamara D, Cavadino A, Alsweiler JM (2023). Caffeine to prevent intermittent hypoxaemia in late preterm infants: randomised controlled dosage trial. Arch Dis Child Fetal Neonatal Ed.

[33] Schmidt B, Roberts RS, Davis P, Doyle LW, Barrington K, Ohlsson A (2006). Caffeine therapy for apnea of prematurity. NEJM.

[34] Iranpour R, Armanian AM, Miladi N, Feizi A (2022). Effect of prophylactic caffeine on noninvasive respiratory support in preterm neonates weighing 1250–2000 g: a randomized controlled trial. Arch Iran Med.

[35] Armanian A-M, Iranpour R, Faghihian E, Salehimehr N (2016). Caffeine administration to prevent apnea in very premature infants. Pediatr Neonatol.

[36] Steer P, Flenady V, Shearman A, Charles B, Gray PH, Henderson-Smart D (2004). High dose caffeine citrate for extubation of preterm infants: a randomised controlled trial. Arch Dis Child Fetal Neonatal Ed.

[37] Schmidt B, Anderson PJ, Doyle LW, Dewey D, Grunau RE, Asztalos EV (2012). Survival without disability to age 5 years after neonatal caffeine therapy for apnea of prematurity. JAMA.

[38] Schmidt B, Roberts RS, Anderson PJ, Asztalos EV, Costantini L, Davis P (2017). Academic performance, motor function, and behavior 11 years after neonatal caffeine citrate therapy for apnea of prematurity. JAMA Pediatr.

[39] Vliegenthart R, Miedema M, Hutten GJ, Van Kaam AH, Onland W (2018). High versus standard dose caffeine for apnoea: a systematic review. Arch Dis Child Fetal Neonatal Ed.

[40] Brattström P, Russo C, Ley D, Bruschettini M (2018). High-versus low-dose caffeine in preterm infants: a systematic review and meta-analysis. Acta Paediatr.

[41] Bruschettini M, Brattström P, Russo C, Onland W, Davis P, Soll R. Caffeine dosing regimens in preterm infants with or at risk for apnea of prematurity. Cochrane Database Syst Rev. 2023;2023. 10.1002/14651858.CD013873.PUB2. 10.1002/14651858.CD013873.pub2 PMC10089673 37040532

[42] Henderson-Smart DJ, De Paoli AG. Prophylactic methylxanthine for prevention of apnoea in preterm infants. Cochrane Database Syst Rev. 2010. 10.1002/14651858.CD000432.pub2. 10.1002/14651858.CD000432.pub2 PMC7032541 21154344

[43] Marques KA, Bruschettini M, Roehr CC, Davis PG, Fiander M, Soll R. Methylxanthine for the prevention and treatment of apnea in preterm infants. Cochrane Database Syst Rev. 2023;2023. 10.1002/14651858.CD013830.PUB2. 10.1002/14651858.CD013830.pub2 PMC10617014 37905735

[44] Chen J, Jin L, Chen X. Efficacy and safety of different maintenance doses of caffeine citrate for treatment of apnea in premature infants: a systematic review and meta-analysis. Biomed Res Int. 2018;9061234. 10.1155/2018/9061234. 10.1155/2018/9061234 PMC6323495 30671477

[45] Schmidt B, Roberts RS, Davis P, Doyle LW, Barrington K, Ohlsson A (2007). Long-term effects of caffeine therapy for apnea of prematurity. NEJM.

[46] Moschino L, Zivanovic S, Hartley C, Trevisanuto D, Baraldi E, Roehr CC (2020). Caffeine in preterm infants: where are we in 2020?. ERJ Open Res.

[47] Erenberg A, Leff R, Wynne B (1998). Results of the first double blind placebo (pl) controlled study of caffeine citrate (cc) for the treatment of apnea of prematurity (AOP). Pediatr Res.

[48] Doyle LW, Ranganathan S, Cheong JL (2017). Neonatal caffeine treatment and respiratory function at 11 years in children under 1,251 g at birth. Am J Respir Crit Care Med.

[49] Mürner-Lavanchy IM, Doyle LW, Schmidt B, Roberts RS, Asztalos EV, Costantini L (2018). Neurobehavioral outcomes 11 years after neonatal caffeine therapy for apnea of prematurity. Pediatrics.

[50] Schmidt B, Anderson PJ, Asztalos EV, Doyle LW, Grunau RE, Moddemann D (2019). Self-reported quality of life at middle school age in survivors of very preterm birth: results from the caffeine for apnea of prematurity trial. JAMA Pediatr.

[51] Doyle LW, Schmidt B, Anderson PJ, Davis P, Moddemann D, Grunau RE (2014). Reduction in developmental coordination disorder with neonatal caffeine therapy. J Pediatr.

[52] Oliphant EA, McKinlay CJD, McNamara DG, Alsweiler JM (2020). Caffeine prophylaxis to improve intermittent hypoxaemia in infants born late preterm: a randomised controlled dosage trial (Latte Dosage Trial). BMJ Open.

[53] Gray PH, Flenady VJ, Charles BG, Steer PA (2011). Caffeine citrate for very preterm infants: effects on development, temperament and behaviour. J Paediatr Child Health.

